# Effect of Carbon Nanotube-Metal Hybrid Particle Exposure to Freshwater Algae Chlamydomonas reinhardtii

**DOI:** 10.1038/s41598-018-33674-7

**Published:** 2018-10-17

**Authors:** Worawit Intrchom, Megha Thakkar, Raymond F. Hamilton, Andrij Holian, Somenath Mitra

**Affiliations:** 1 Department of Chemistry and Environmental Science, New Jersey Institute of Technology, Newark, NJ 07102 USA; 2 Department of Biomedical and Pharmaceutical Sciences, Center for Environmental Health Sciences, University of Montana, Missoula, MT 59812 USA

## Abstract

We demonstrate for the first time the toxicity of carbon nanotube (CNT) metal hybrids on freshwater algae. Carbon nanotube-silver (CNT-Ag) and platinum hybrids (CNT-Pt) were synthesized and exposed to Chlamydomonas reinhardtii *(C*. *reinhardtii*), and their toxicity was compared to the pure metal salts. Interactions between CNT-metal and algae were studied using electron microscopy and it was observed that while outer membrane of the algal cell was damaged as a result of Ag^+^ toxicity from pure Ag, the CNT-Ag only caused the distortion of the cell wall. It was also observed that the CNT-Ag particles could be internalized and enclosed in internal vesicles in the algal cells. Long-term exposure of the CNT-metals showed delay in algal growth. CNT-Ag at a concentration of 5.0 mg/L showed 90% growth inhibition and also showed a significant effect on photosynthetic yield with a 21% drop compared to the control. It was observed that pure silver was more toxic compared with CNT-Ag for both growth and photosynthesis in the 96-hour exposure. In general, CNT-Pt showed significantly less toxic effects on the algae than CNT-Ag. Based on this study, it is postulated that the CNT suppressed the release of Ag^+^ from CNT-Ag hybrids, thus reducing overall toxicity.

## Introduction

Due to the extraordinary chemical, physical, mechanical and electronic properties, the production of engineered nanoparticles, including CNT is increasing at a dramatic rate. Applications of CNT include touch screens, solar cells, batteries, super capacitors, organic light emitting diodes, flat screen displays, sensors, and fuel cells^1^. CNT are also used as support materials where metals, metal oxides, polymers and other materials are immobilized. Examples of metal immobilization include Au, Ag, Pt, Ru, Pd, and Co to name a few. For instance, silver nanoparticles (AgNP) have been used in diverse disinfection, water treatment and other applications^2–6^, and CNT-Ag composites are being employed as antimicrobial agents, sensing materials, catalysts and sorbents^7–10^. Similarly, Pt has been functionalized on CNT to serve as catalysts for organic synthesis and as electrocatalysts in fuel cells^11–14^. The applications of such CNT-metal hybrids are expected to expand because immobilization on the CNT ensures nano structuring of the metal for high activity, and it also reduces the amount of expensive metals needed for a specific application.

Like all other pollutants, at the end of life cycle the CNT-metal hybrids are expected to end up in the environment^15^. This increases their public health and ecological risks^16^. The released CNT-metal hybrids may eventually migrate and deposit in water resources that act as sinks for the pollutants, and then affect living organisms through the aquatic food web^17,18^. In the freshwater ecosystem, algae play an important role as the dominant primary producers that convert inorganic carbon into organic matter and provides biomass for the food web^19^. As a result, algae are chosen as an initial biological indicator for environmental pollution monitoring, and used as a test organism in ecotoxicity of nanomaterials^19,20^.

AgNP and CNT have been examined for their toxicity to different living organisms such as bacteria, algae, fish and mice^21–29^. The results of algal toxicity studies have shown that silver toxicity was primarily due to the release of silver ions and its derivatives^26^. Mechanism of Ag toxicity in the green algae *C*. *reinhardtii* is known to be from internalization of Ag^+^, but both forms of silver (AgNP and Ag^+^) are toxic to this microorganism^27^. A few studies on the effects of platinum nanoparticles (PtNP) on aquatic organisms, including algae have been reported^30–32^. A recent study concluded that a shading effect and oxidative stress are two main causes of the PtNP toxicity^32^. Toxicity of different kinds of CNTs including carboxylated single walled carbon nanotubes, pristine multiwalled carbon nanotubes, carboxylated multiwalled carbon nanotubes, and multiwalled carbon nanotubes with diuron has been studied on algae^21,23,24,28,29^, and the results show that CNT can affect the growth rate, photosynthesis capacity, and generate oxidative stress^28,29^. However, the toxicity of CNT-metal hybrids such as CNT-Ag and CNT-Pt are unknown.

The objective of this research is to study the toxicity of CNT-Metal composites on freshwater algae *C*. *reinhardtii*. Of particular interests are CNT-Ag and CNT-Pt, both of which have huge commercial potential. Long term (7 days) toxicity of CNT-Ag and CNT-Pt was tested with the algae and compared to pristine-CNT and CNT-COOH. In 96-h toxicity testing, the cultures were exposed to CNT-Ag and CNT-Pt and compare to pure metals. Lastly, the interaction of CNT-metal hybrids and algae was investigated using electron microscopy.

## Results and Discussion

### Characterizations

**Figure 1:**
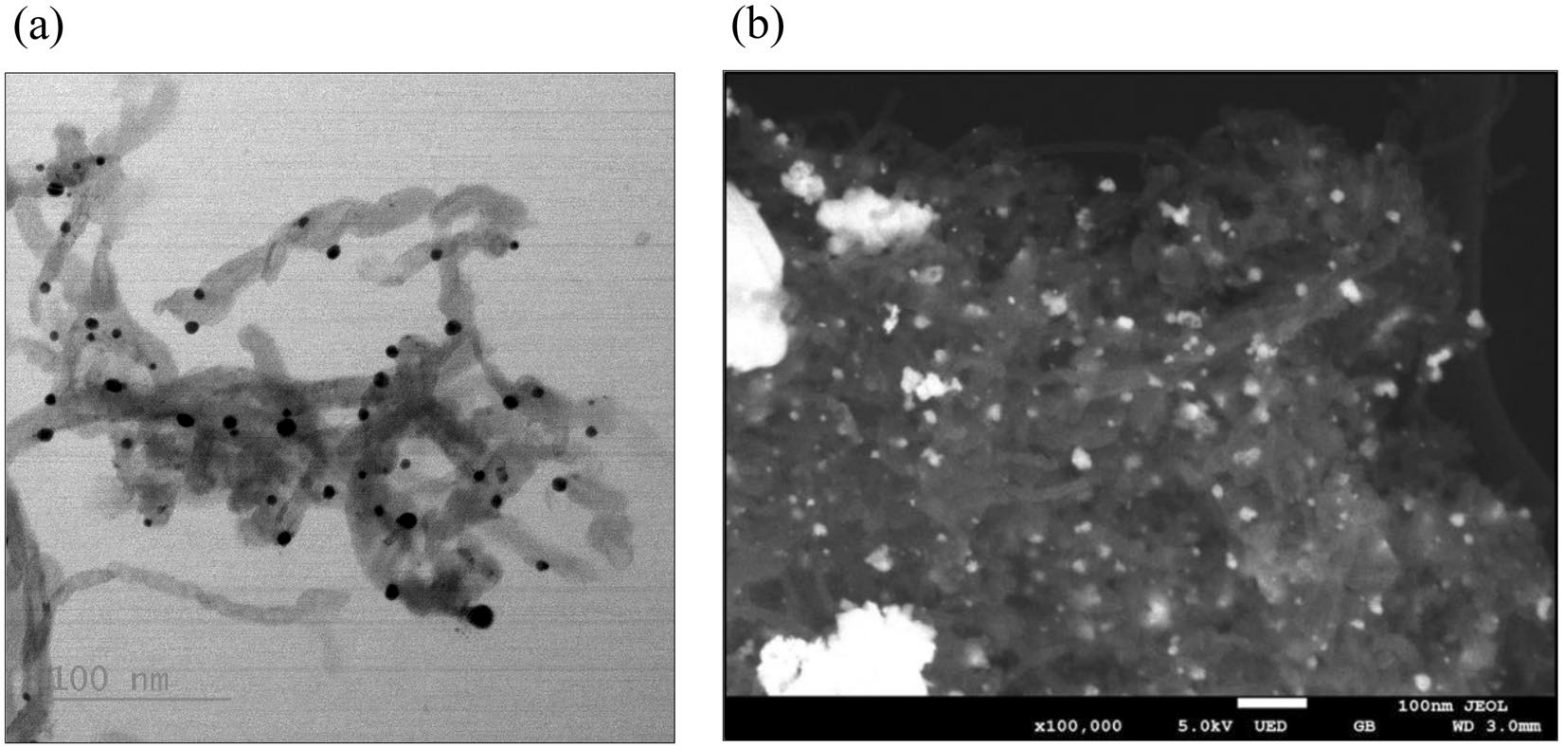
TEM image of (**a**) CNT-Ag, and SEM image of (**b**) CNT-Pt.

**Figure 2:**
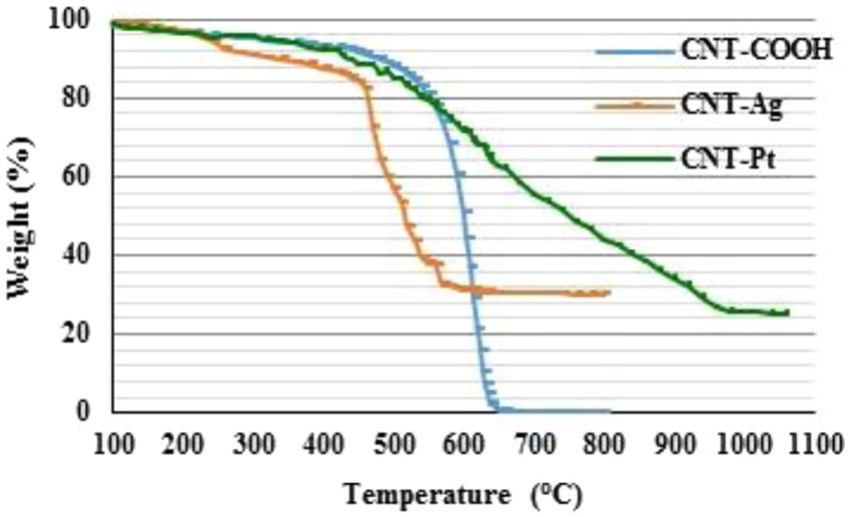
TGA data for CNT-COOH, CNT-Ag, and CNT-Pt.

The presence of metals on CNT hybrids are seen from the SEM and TEM images (Fig. 1) and were confirmed by EDS. The dark spots in the bright field TEM image show the deposition of Ag on CNT (Fig. 1a) and the white spots on SEM image show Pt on CNT (Fig. 1b), and EDS data indicated two main elements of the CNT-Ag and CNT-Pt hybrids. The length and diameter of the CNTs were 2.6 ± 0.8 µm and 20–30 nm respectively, while the size ranges of the Ag and Pt particles based on TEM and SEM measurements were found to be between 4.8–15.5 nm and 6.1–55.2 nm. The amount of Ag and Pt on CNT was quantified by TGA. As shown in Fig. 2, the resulting weight above 600 °C and 900 °C was attributed to the weight of residual nano metals, the quantity of Ag and Pt on the surface of CNT was found to be around 30 and 25%, respectively. The quantity of Ag and Pt leached from CNT-metal hybrid into the culture medium was below the detection limit of ICP-MS measurement which were 0.03 and 0.1 ppb for Ag and Pt, respectively. However, when the equivalent amounts of 0.15 and 1.5 mg/L of AgNO_3_ and 0.125 and 1.25 mg/L of PtCl_2_ were added to the culture, they could be detected quantitatively. This is implied that leaching of metal ions from CNT-Ag and CNT-Pt hybrid were minimal.

### Toxicity Testing

#### Long term toxicity testing of CNT-metals on C. reinhardtii

**Figure 3:**
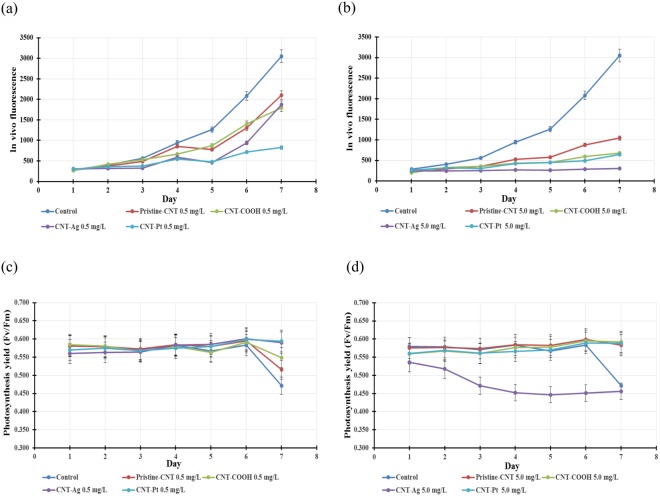
Growth curves of *C*. *reinhardtii* exposed to pristine and functionalized CNT. at concentrations of (**a**) 0.5 mg/L and (**b**) 5.0 mg/L. The photosystem II (PSII) quantum yield efficiency (Fv/Fm) of *C*. *reinhardtii* exposed to pristine and functionalized-CNT at concentrations of (**c**) 0.5 mg/L and (**d**) 5.0 mg/L.

Algal cells were inoculated into Bold’s basic medium with the initial concentrations of 0.5 and 5.0 mg/L of pristine-CNT, CNT-COOH, CNT-Pt and CNT-Ag and *in vivo* fluorescence was measured for 7 days. To study the toxicity of these materials on the growth of algae, the exponential growth phase of algae was monitored and are shown in Fig. 3.

Results from 7-day exposure showed that pristine-CNT and CNT-metal composites delayed the growth of algae. However, pristine-CNT and CNT-COOH at the concentration of 0.5 mg/L had insignificant effects on algae growth, compared to control culture (*P* > 0.05). In contrast, CNT-Pt significantly affected the growth of algae with 73% growth inhibition and showed increased toxic effects over CNT-Ag at this concentration as shown in Fig. 3a.

At a concentration of 5.0 mg/L, all forms of CNT significantly caused the delay of algal growth and CNT-Ag had the highest impact on the growth of algae, followed by CNT-Pt with 90% and 79% growth inhibition respectively, compared to the control sample, as shown in Fig. 3b.

The effect of pristine-CNT and functionalized CNT on the photochemical processes in algae was also determined. The photosystem II (PSII) quantum yield efficiency (Fv/Fm) was analyzed using single turnover flash fluorescence induction and relaxation (FIRe). The results show that Fv/Fm at 5.0 mg/L CNT-Ag was significantly lower than the control and other concentrations of CNT. The Fv/Fm value of control culture and most of other cultures were quite stable and reached the maximum value (0.583–0.601) at day 6 and trended to decrease (Fig. 3(c,d)), while the Fv/Fm value at 5.0 mg/L of CNT-Ag decreased and reached the minimum value (0.447) at day 5 and then trended to increase slightly afterwards as illustrated in Fig. 3d.

The results indicate that pristine-CNT, CNT-COOH and composite CNT-metals have adverse effects on the growth of algae in a concentration dependent manner, the higher concentration led to more delay in algal growth. However, after a period of time, the growth curve of cultures with low concentration of tested materials trended to gradually increase, while the cultures with high concentration of CNT-Pt and CNT-Ag induced very low algal growth rates. This suggests that the pristine CNT and CNT-metal hybrid caused temporary growth inhibition effects on the algae, after which they tended to recover. This has been attributed to the decrease in toxicity of the nano particles with time, which are associated with particle aggregation and the algal secretions (described in the following section) that reduced the net reactive components as well as the active surface area of the nano particles^33^. Similar growth recovery phenomena were also reported in toxicity studies of TiO_2_ and CdTe quantum dots with *C*. *reinhardtii*^33^ and our previous study of cytotoxic effects of CNT-COOH on marine alga *D*. *tertiolecta*^28^.

Photosynthesis yield not only reflects the changes of algal photosynthetic activity, but is also related to the changes of cultivation conditions, growth and the state of algae^34^. Our study showed that the quantum yield of PS II was not significantly affected by any CNT composite nanoparticles at a concentration of 0.5 mg/L. However, the Fv/Fm values of control, pristine-CNT, and CNT-COOH cultures declined after day 6. The drop of the Fv/Fm values is attributed to the change in the culture environment and the state of the algae^35^. The increase in algal mass or the transition of algal growth state from lag to exponential phase shown by growth curve, and these may alter the environment and lead to a drop in Fv/Fm. At a concentration of 5.0 mg/L, the photosynthesis yield was reduced only in the presence of CNT-Ag. The effect on algal photosynthesis is also known to be caused by shading effects of nanoparticles, including CNT^28,36–38^. In our study, among the different CNT forms studied, CNT-Ag showed the maximum effect during the interaction with the algae and this is in line with the inhibition of photosynthesis observed here.

#### 96-h toxicity testing for CNT-metals on C. reinhardtii

**Figure 4:**
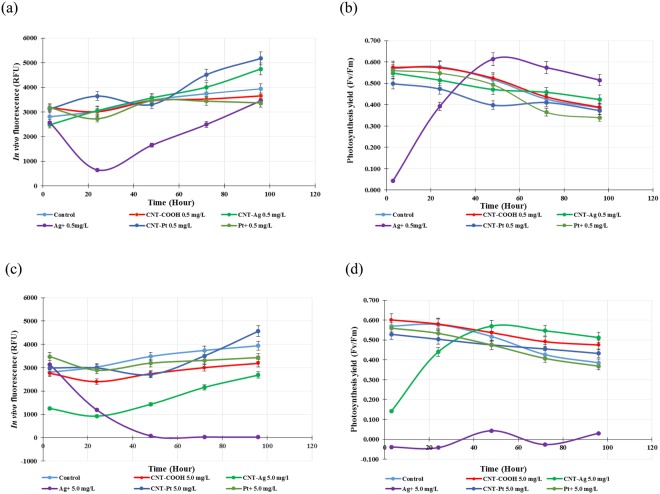
Effects of metal functionalized CNT and metal ions at concentrations of 0.5 (**a**) and 5.0 (**c**) mg/L on *in vivo* fluorescence. Effects of metal functionalized CNT and metal ions at concentrations of 0.5 (**b**) and 5.0 (**d**) mg/Lon the photosystem II (PSII) quantum yield efficiency (Fv/Fm) of *C*. *reinhardtii*.

At the concentration of 0.5 mg/L, only Ag^+^ had significant impact on the algal growth rate (*P* < 0.05), while CNT-COOH, CNT-Pt, CNT-Ag and Pt^+^ had insignificant effects compared to the control (*P* > 0.05). The results also showed that Ag^+^ had the most effect on the growth of algae (Fig. 4a). Studies of the effects on the photosystem II (PSII) quantum yield efficiency (Fv/Fm) of the algal cells, demonstrated that only Ag^+^ showed an adverse effect on photosynthesis during 24-h exposure (*P* < 0.05). However, after 24 h, the growth and the light conversion of this culture trended to improve with time. The amount of *in vivo* fluorescence was close to the other conditions by the end of testing, while Fv/Fm value exceeded that of other cultures at 48 h of testing as shown in Fig. 4b.

At the concentration of 5.0 mg/L, all tested materials showed harmful effects on the growth of algae, while CNT-Ag and Ag^+^ had the most significant effects (*P* < 0.05). This is shown in Fig. 4c and d. During 24-h exposure, the CNT-Ag significantly inhibited algal growth, causing considerable reduction in *in vivo* fluorescence and photosynthesis yield efficiency. However, after 24-h period, the growth curve trended to increase gradually with 32% growth reduction compared to the control sample at 96 h of testing, while Fv/Fm of the culture quickly recovered, and exceeded other cultures and the control, and then tended to reduce like other cultures after 48 h of testing. Whereas, the growth rate of the cultures exposed to Ag^+^ gradually decreased during 48-h exposure, and then reached and leveled off with 99% growth reduction compared to control. Furthermore, Ag^+^ also showed strong effects on algal photosynthesis; Fv/Fm ratio of the samples hovered around zero throughout the 96-h exposure as shown in Fig. 4d.

In summary, CNT-Pt and Pt^+^ did not show significant negative effects on the growth of algae (*P* > 0.05). The growth and the light conversion process of algae at the concentration of 0.015 mg/L as Ag^+^ and of 5.0 mg/L of CNT-Ag had a similar pattern of growth inhibition; both temporarily inhibited the growth of algae during 24 h of exposure, and after that the growth curves showed recovery. However, AgNO_3_ at the concentration of 0.15 mg/L that is equal to 5.0 mg/L of CNT-Ag as the amount of Ag^+^ inhibited the growth and the light conversion process of algae completely. These results are in line with the observed interactions of CNT-Ag, Ag^+^ with the algae.

It has been reported^39^ that the toxicity of AgNP resulted from the ionic silver released from the particles and it has also been suggested^26^ that ionic silver release can be enhanced due to its active dissolution upon contact with the algae. In our study, it can be concluded that Ag^+^ had higher toxic effects than the CNT-Ag composite and it seemed that CNT assisted in suppressing the release of ionic silver from the CNT-Ag composite material. The negatively charged carboxylated surface can attract silver ions and prevent them from being released into the media. The electron clouds on the CNT surface also provide a mild attractive force between the nanotubes and Ag^+ ^^40^. In addition, some reducing agents may reverse the ion release reaction. These include groups containing C=C, hydroxylic functionality, and the oxidation debris that may be present on the CNT surface^41^, which are known to be structurally similar to organic acids^42,43^. Finally, carboxyl groups on functionalized CNT exist in the form of COO^−^ in aqueous media, which serve as a buffer and combine with H^+^ to form COOH, resulting in a lower H^+^ concentration and hence could inhibit the silver oxidation reaction as illustrated in the equation below.

1 $$2{{\rm{Ag}}}_{({\rm{s}})}+1/2{{\rm{O}}}_{2({\rm{aq}})}+2{{{\rm{H}}}^{+}}_{({\rm{aq}})}\iff 2{{\rm{Ag}}}^{+}({\rm{aq}})+{{\rm{H}}}_{2}{\rm{O}}$$

In general, it is likely that the toxicity of silver composites depends not only on their existing forms, but also surface functionalization, temperature and media.

### Interaction of CNT-metals hybrids with *C. reinhardtii*

**Figure 5:**
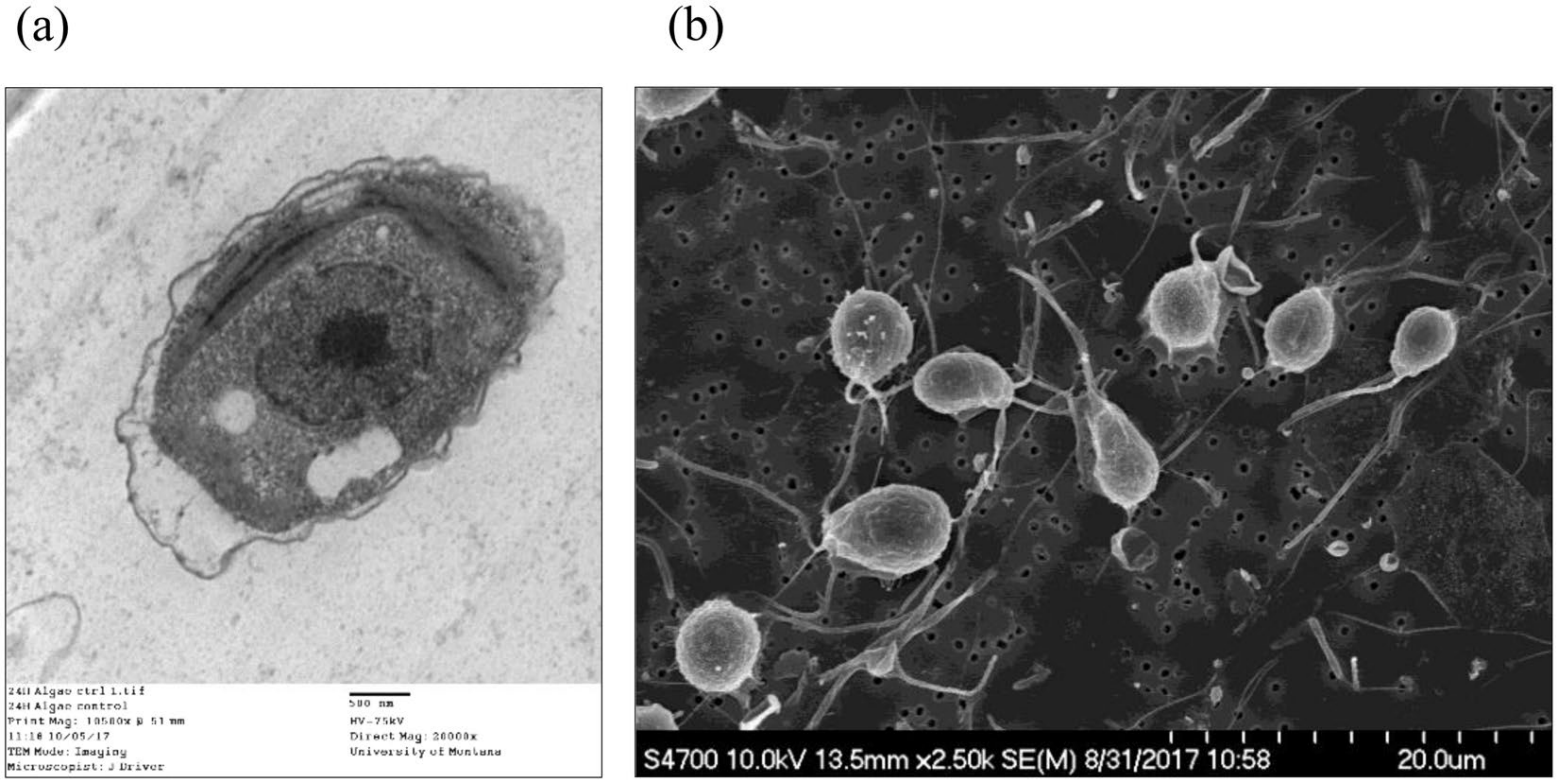
TEM image (**a**) and SEM image (**b**) of algae of control culture.

The interaction of CNT-metal hybrids and algae was investigated using TEM and SEM. Figure 5a,b show the TEM and SEM image of the unicellular green macroalgae from control cultures. The TEM image presented is a single algal cell with its organelles surrounded by a cell wall, while the SEM image illustrates the distribution of the flagellate algal cells before being exposed to the tested materials.

**Figure 6:**
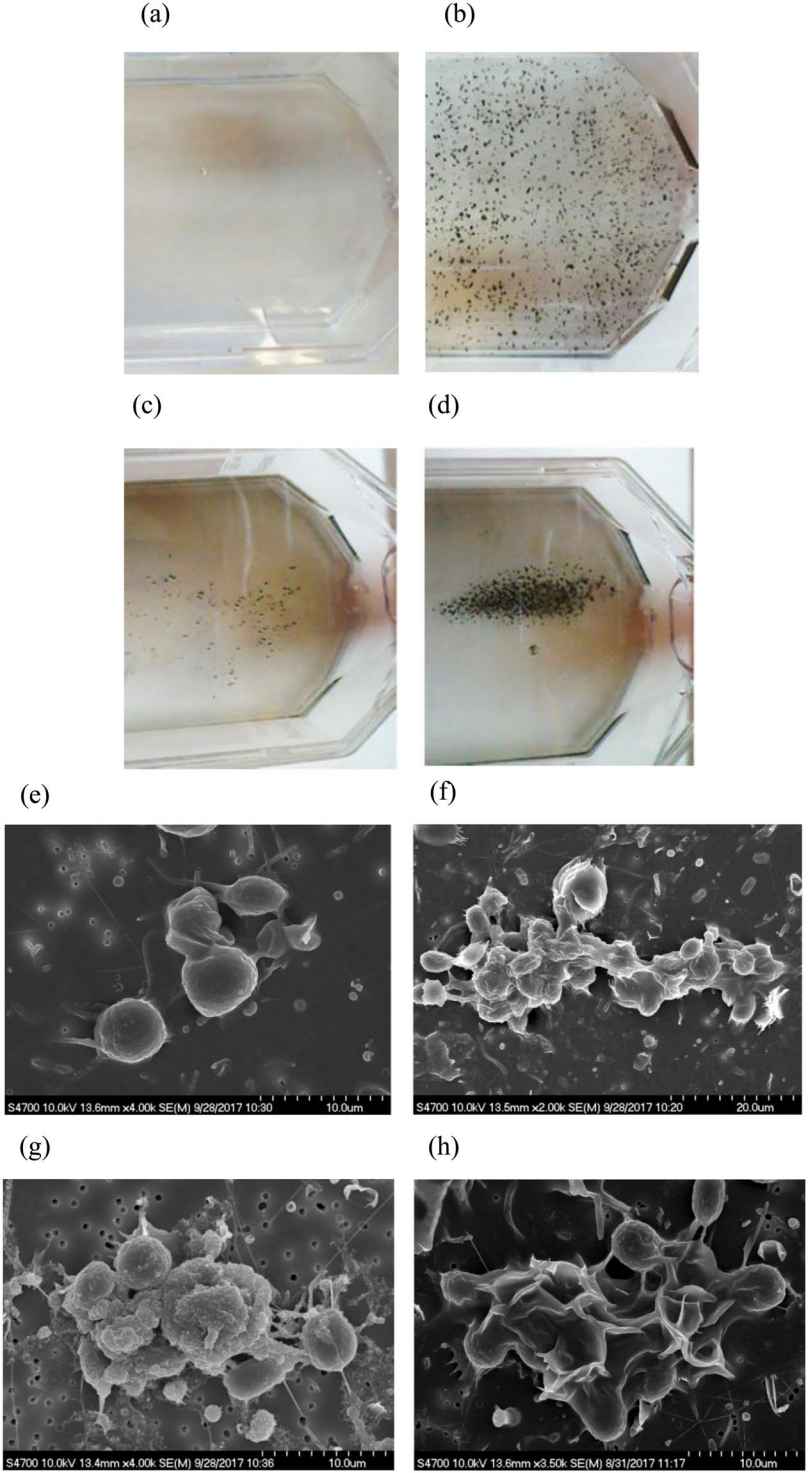
Macro-appearance of algae cultures indicating aggregate clumping of algae with CNT variants after 3 hours. (**a**) Control culture with algae alone. (**b**) Algae cultured with 25 µg/mL CNT-COOH. (**c**) Algae cultured with 25 µg/mL CNT-Ag. (**d**) Algae cultured with 25 µg/mL CNT-Pt. Representative SEM images of algae interactions with CNT variants 3 hours post-exposure indicating particle/cell aggregation. (**e**) Control culture with algae alone. (**f**) Algae cultured with 25 µg/mL CNT-COOH. (**g**) Algae cultured with 25 µg/mL CNT-Ag. (**h**) Algae cultured with 25 µg/mL CNT-Pt.

Following CNT introduction, the algae cultures showed visible aggregation, regardless of the CNT variant. The CNT particles aggregated around algae indicating strong interactions with the latter. This is evident from Fig. 6b–d where the aggregation pattern depended upon the CNT form. No separate precipitation of the CNT was observed. After 3-h exposure, the CNT-Pt caused the most aggregation (Fig. 6d). Algae/particle aggregation was confirmed with SEM (Fig. 6(f–h)). The algal cell wall is essentially a polymer that can be easily reshaped depending on the environmental situation^44–47^. Secretion of the extracellular polymeric substance is also known as a protective mechanism of organisms against toxicity. Similar responses have been reported from algae and bacteria against nanoparticles such as CuO and Ag nanoparticles^28,39,48,49^. The most extreme reaction was seen in algae exposed to CNT-Ag (Fig. 6g). In this case, the algal surface cell wall appearance was very ruffled, indicative of a stress response similar to high salt exposure; H_2_O_2_ – induced cell death or desiccation^50–52^. The depolarization of the cell wall may indicate the occurrence of reactive oxygen species (ROS) production and oxidative stress when cells contact nanoparticles. Both CNT and nanosilver are well known that are able to induce oxidative stress through ROS independent mechanisms^53,54^. Studies have shown that Ag nanoparticles are toxic to algae, algae reproduction and microorganisms in general^55,56^. Algae also responded to the CNT-COOH, but the distortions in the cell wall were less apparent (Fig. 6f) and similar to the control culture (Fig. 6e). The CNT-Pt caused the algal cell wall to appear virtually smooth (Fig. 6h), almost opposite of the CNT-Ag effect. Overall, the CNT-Pt did not appear to be as toxic.

**Figure 7:**
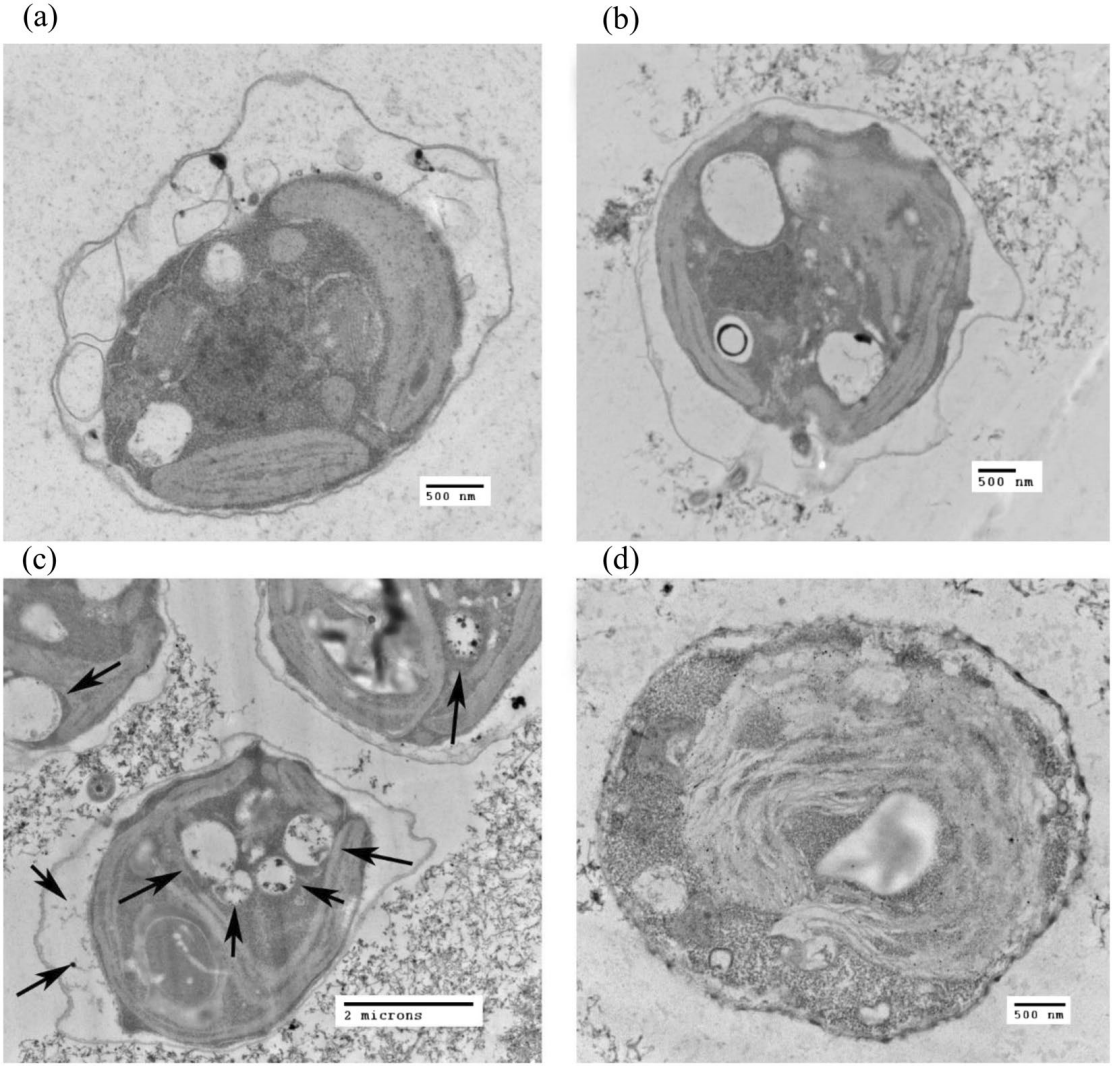
Representative TEM images of algae interactions with CNT variants 24 hours post-exposure. (**a**) Control culture with algae alone. (**b**) Algae cultured with 25 µg/mL CNT-COOH. (**c**) Algae cultured with 25 µg/mL CNT-Ag. (**d**) Algae cultured with 25 µg/mL CNT-Pt. Black arrows indicate particle internalization featuring extensive vacuole formation in algae.

After 24-h exposure, TEM was utilized to monitor the effects of CNT-metal hybrids and are shown in Fig. 7. Algae particle internalization was limited or nonexistent in the case of CNT-COOH (Fig. 7b) and CNT-Pt (Fig. 7d). However, internalization was observed in the case of CNT-Ag, since a number of particle-containing vacuoles in each algae exposed to the CNT-Ag were observed (Fig. 7c). The internalization of CNT into the algae has also been shown in other reports^57,58^, especially in the presence of the surfactants that increase the hydrophobicity of algal cell surface and enhance the chances for cell uptake^58^. In our study, the presence of Ag may have altered the cell membrane to allow CNT-Ag internalization. This could account for the increased toxicity of the CNT-Ag contrasted with the other CNT variants. The uptake mechanism of algae for carbon particles is not well understood, although clathrin has been isolated from algae and this protein is a constituent of basic cellular internalization^59^, and simple endocytosis is believed to be an evolution ancient trait in plants^60^. This fact, combined with the greater internalization of CNT-Ag most likely accounts for the toxicity, although the differential mechanism of uptake still needs to be determined.

**Figure 8:**
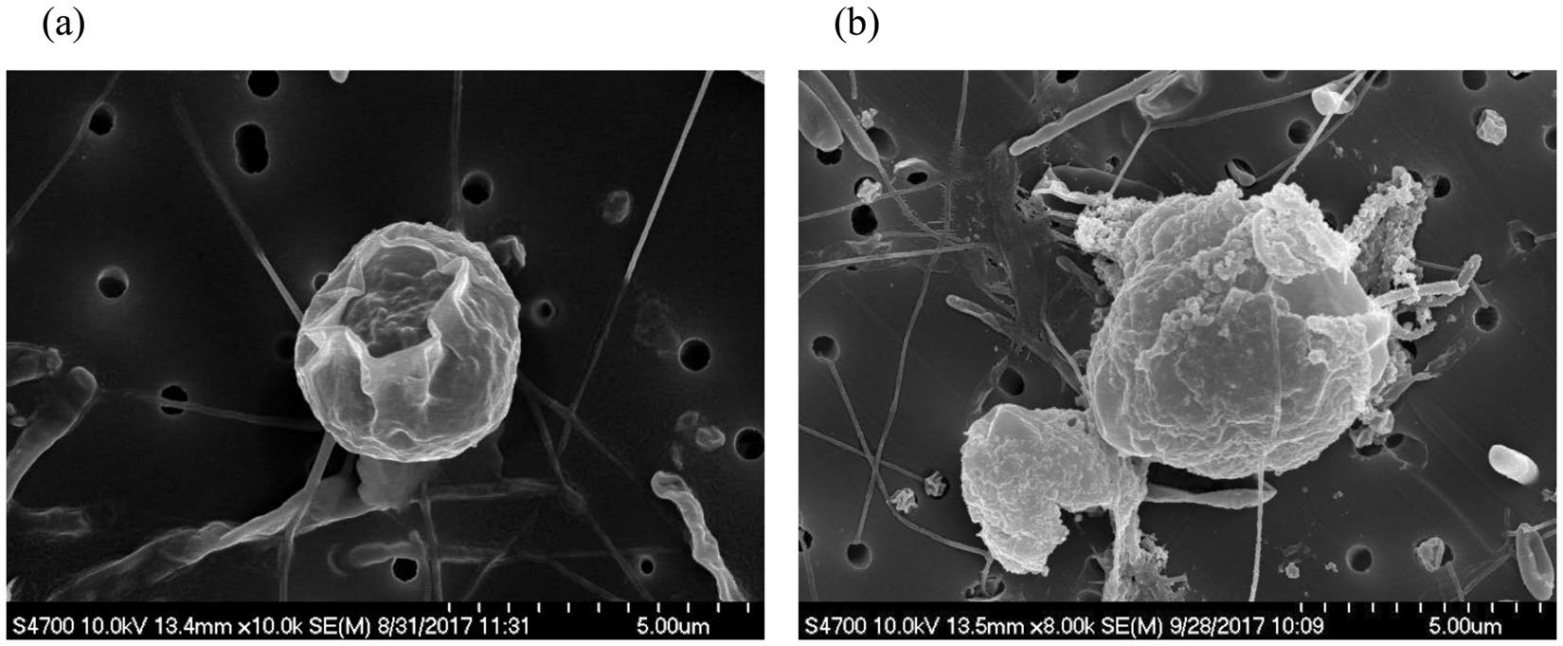
Algal cells after 3-h (**a**) and 24-h (**b**) exposure to Ag^+^.

The results of the interactions between CNT-Ag and algae using TEM showed that CNT-Ag led to some effects on algal activities; growth and photosynthesis. These effects were further supported by growth (*in vivo* fluorescence) data and photosystem II (PSII) quantum yield data as mentioned in the previous section. Unlike CNT-Ag, Ag^+^ as AgNO_3_ was very toxic to the algae and caused the algae to die within a few hours. SEM images exhibited the algal cell after 3 and 24-h exposure to Ag^+^ (Fig. 8). After 3-h exposure, SEM image showed that the outer membrane of the algal cell was damaged as a result of Ag^+^ toxicity (Fig. 8a), while Fig. 8b illustrates what is consistent with deterioration and degradation of the cell wall after 24 h of exposure. The mechanisms of Ag^+^ toxicity on algae may result of the induction of oxidative stress and cell membrane damage at the cell surface or oxidative stress through ROS generation inside the algal cell^54^ or binding and denaturation of proteins.

## Conclusion

Carbon nanotube-metal hybrids CNT-Ag and CNT-Pt affected growth and photosynthesis activities of freshwater green algae *C*.*reinhardtii*. For long-term exposure, all forms of CNT caused the delay of algal growth. During seven day exposure, CNT-Ag at a concentration of 5.0 mg/L had the highest impact with 90% growth inhibition, and affected the photosynthetic yield (fv/fm) with the minimum value of 0.45. In short-term 96 hour testing, only high concentrations of CNT-Ag (5.0 mg/L) had significant effects on the activities of exponentially growing algal cells. The algal activities recovered after 24 h. With the same amount of Ag as AgNO_3_, the behavior was quite different. AgNO_3_ completely inhibited the growth and the light conversion process of algae, it caused damage to the cell membrane and the algae died within a few hours. It is concluded that CNT play a role in suppressing the release of ionic silver from the CNT-Ag hybrid and thus reduced toxicity.

## Materials and Methods

### Preparation of CNT-Ag and CNT-Pt

Multiwall carbon nanotubes (OD 20–30 nm, length 10–30 µm, purity >95%) were purchased from Cheap Tubes Inc., and all other chemicals were purchased from Sigma Aldrich with purity higher than 95%. First, the CNT-COOH were prepared by microwave induced reaction in a Microwave Accelerated Reaction System (CEM Mars) according to procedures published before^61^. In this process, seven microwave reaction vessels were used, each containing 1000 mg pristine multiwall carbon nanotubes (pristine-CNT) and 40 mL of 1:1 concentrated H_2_SO_4_ and HNO_3_. The reaction vessels were subjected to microwave radiation with the temperature set at 140 °C for 20 min. After the reaction, the reactants were transferred into a beaker with Milli-Q water and cooled down to room temperature. The product was filtered under vacuum using a Teflon membrane with a pore size of 0.45 µm. The resulting solids were thoroughly washed with Milli-Q water to a neutral pH and then dried in a vacuum oven at 50 °C for 24 h. The degree of carboxylation was quantified based on the C:O molar ratio which was found to be 11:1 and in line with EDX data which showed 14.3% by weight. This C:O ratio was in line with our previous publications^62,63^.

The CNT-COOH were further functionalized with silver and platinum in a microwave reactor following the procedure published before^64^. For metal functionalization, 20 mg of CNT-COOH and 10 mL of Milli-Q water were sonicated for 5 min. To this was added 10 mg of AgNO_3_ or PtCl_2_ and 30 mL of ethylene glycol. After 10 min sonication, the mixture was subjected to microwave radiation at 100 °C for 5 min. After the reaction, the reactants were transferred into a beaker with Milli-Q water and cooled to room temperature. The product was filtered under vacuum using a 0.45 µm Teflon membrane and the resulting solids were thoroughly washed with Milli-Q water until a neutral pH was reached, and then dried in a vacuum oven at 50 °C for 24 h.

Images of CNT hybrid materials were produced by transmission electron microscopy (TEM, JEOL; model JEM-2800F), and scanning electron microscopy (SEM, JEOL; model JSM-7800F) with elemental analysis done by using an energy dispersive X-ray analyzer (EDS). The amount of metals on CNT was confirmed by thermo gravimetric analysis (TGA) performed under a flow of 10 ml/min air and heated from 20 to 800 °C for CNT-Ag and from 20 to 1,100 °C for CNT-Pt at a heating rate of 10 °C per min. The amount of Ag in CNT-Ag and Pt in CNT-Pt were found to be 30 and 25% by weight, respectively. The quantity of Ag and Pt released from CNT-Ag and CNT-Pt were determined by Inductively Coupled Plasma-Mass Spectrometer (ICP-MS, Agilent; model 7900).

### Algal strain and culturing and Exposure to CNT-Metals

Unicellular green alga, *C*. *reinhardtii* (152040), was obtained from the Carolina Biological Supply Company. Algae were cultured in Bold’s basic medium in acid-cleaned 250 mL polycarbonate bottles (Nalgene) in a growth chamber with 120 µEm^−2^ s^−1^ illuminations, 12 h:12 h light–dark cycle and 19 ± 0.1 °C.

In exposure experiments, long term and 96-h exposure were carried out. In the first study, algae were exposed to pristine-CNT, CNT-COOH, CNT-Pt and CNT-Ag by inoculating a small amount of algal culture to Bold’s basic medium (1:10, v/v), which had been pre-equilibrated with 0.5 and 5 mg/L of each CNT for 3 h. The toxicity of pristine-CNT and each functionalized CNT on algae were assessed on a daily basis. In this study, the nanohybrids was in the media for 7 days.

In the second study, algae were inoculated with Bold’s basic medium with the ratio of 1: 10 (v/v) and cultured in a growth chamber until growth reached the exponential phase. In this phase, the cultures were exposed to CNT-Ag and CNT-Pt at concentrations of 0.5 and 5.0 mg/L by adding the CNT-metals stock suspensions directly into the cultures. To compare the toxicity of CNT-Ag and CNT-Pt with pure metals, AgNO_3_ with the concentrations of 0.15 and 1.5 mg/L of Ag (to simulate CNT-Ag) and PtCl_2_ with the concentrations of 0.125 and 1.25 mg/L (to simulate CNT-Pt) at the same concentration as the amount of the respective metals immobilized on the CNT were examined for their toxicity. In these experiments, the nanohybrids was in the media for 96 h and the toxicity effects were assessed on a daily basis for 96 h. Control cultures (cultures without CNT addition) and culture blanks (media without algae inoculation) were set up in parallel with the exposure treatments. Cultures were conducted in triplicates in both studies.

### Toxicity assessment

The toxic effects of CNT-Ag and CNT-Pt exposure on algal growth and photosynthesis were assessed on a daily basis. Algal growth was monitored by *in vivo* fluorescence using a Turner Designs’ Trilogy Fluorometer, which also had a capability of *in vivo* chlorophyll a measurement (excitation 485 nm; emission 685 nm with a bandwidth of 50 nm). To study the background fluorescent from CNT hybrids, 5 mg/l of the hybrid was added to the algal media without the algae and its fluorescence was measured daily for 5 days. The fluorescence was found to be in the range of 5–19 RFU, which was orders of magnitude lower than what was observed during the experiment. This has also been reported in our previous papers^28,29^. The result showed that the fluorescence from CNT did not contribute significantly to the fluorescence.

Algal photosynthesis was studied with a Fluorescence Induction and Relaxation Fluorometer (FIRe, Satlantic Inc) using single turnover flash protocols^65–67^. The FIRe measurement consisted of an induction and a relaxation phase. In the induction phase, a short 100 µs pulse referred to as a single turnover flash was applied to cumulatively saturate photosystem II (PSII), and the fluorescence induction kinetics from Fo (minimum fluorescence) to Fm (maximum fluorescence) was measured. The FIRe data were processed with FIRe Pro (version 1.3.1) software to obtain the various parameters describing PSII photochemical processes. The maximum quantum yield of PSII photochemistry (Fv/Fm) was obtained from the induction phase. FIRe data were processed with and without background subtraction from the culture blanks.

### Interaction of CNT-metals hybrids with algae

To investigate the toxicity mechanisms, the control culture (algae without CNT) and experimental cultures (algae with CNT-metal composite materials or pure metals) were examined by focusing on the physical interaction between tested materials and algal cells. TEM (Hitachi; model H-7100) and FESEM (Hitachi; model S-4700) were used to view the interaction between the algal cells and the tested materials. First, the algae were maintained in Bold’s media (Sigma, St Louis, MO) for several weeks in large 150 cm^2^ flasks at room temperature, next to a lab window to approximate the necessary 12 h light dark cycle. Then, small algae aliquots (approximately 10^6^ cells/mL) were periodically isolated by centrifugation (200 × g for 5 m), placed in fresh media and exposed to the CNT-Ag, CNT-Pt and Ag^+^ for either 3 or 24 h.

For TEM, the algae were washed once in PBS and resulting algal suspensions were fixed in 2.5% EM grade glutaraldehyde in cacodylate buffer at pH 7.2. The algae were then rinsed in dH_2_O and resuspended in 1% osmium tetroxide for 1 h and rinsed in dH_2_O. The algae were dried in a graded ethanol series followed by embedding of the cell pellet in epoxy. Thin sections were stained with 2% uranyl acetate for 30 min at room temperature, rinsed in dH_2_O, and stained for 5 minutes with Reynolds lead citrate stain. The cells were imaged in TEM operating at 75 kV. For SEM imaging, algal samples were placed in 1% glutaraldehyde in cacodylate buffer, pH 7.2 overnight at 4 °C. A subsample in fixative was also placed in 1% osmium tetroxide 30 min before mounting for FESEM. The glutaraldehyde-fixed and Osmium-treated samples were placed on a 0.1 um pore PVDF filter by syringe filtration followed by an ethanol dehydration series. Filters in 100% ethanol were then placed in Hexamethyldisilazane (HMDS) for a 1-h incubation followed by air-drying in a hood. The dried filter samples were placed on a non-conductive sticky carbon tab on an aluminum stub and sputter coated with gold in a Denton Desk V sputter coater (Denton Vacuum LLV, Moorestown, NJ). Algae samples were imaged in FESEM operating at 10 kV.

The experimental data were analyzed by analysis of variance (ANOVA) using IBM SPSS v. 20 Statistical Software. Probability *P* < 0.05 was accepted as statistically significant. When needed, Tukey’s and Fisher’s multiple comparison *post hoc* corrections, with family error rate of 5% was also used.
